# Foeto-maternal complications associated with low birth weight: A prospective multicenter study in northern Ghana

**DOI:** 10.1371/journal.pone.0266796

**Published:** 2022-04-08

**Authors:** Benjamin Ahenkorah, Samuel Asamoah Sakyi, Gideon Helegbe, Eddie-Williams Owiredu, Linda Ahenkorah Fondjo, Winfred Ofosu, Edmund Muonir Der, Benjamin Amoani, Amma Aboagyewa Larbi, Susanna Cheetham, Priscilla Arthur-Johnson, Grant Jenny Kwablah, Ben Gyan

**Affiliations:** 1 Department of Molecular Medicine, School of Medicine and Dentistry, Kwame Nkrumah University of Science and Technology, Kumasi, Ghana; 2 Department of Medical Laboratory Science, Bolgatanga Technical University, Bolgatanga-Upper East Region, Ghana; 3 School of Medicine and Health Science, University for Development Studies, Tamale, Ghana; 4 Ghana Health Service, Upper East Region, Ghana; 5 Department of Biomedical Sciences, University of Cape Coast, Cape Coast, Ghana; 6 Department of Biochemistry and Biotechnology, College of Science, Kwame Nkrumah University of Science and Technology, Kumasi, Ghana; 7 School of Public Health, Kwame Nkrumah University of Science and Technology, Kumasi, Ghana; 8 School of Medical Sciences, University of Cape Coast, Cape Coast, Ghana; 9 Department of Immunology, Noguchi Memorial Institute for Medical Research, Accra, Ghana; Istanbul Universitesi-Cerrahpasa, TURKEY

## Abstract

**Objective:**

The study evaluated the socio-demographic characteristics, obstetric variables and foeto-maternal complications associated with low birth weight (LBW) in order to provide better treatment and management options.

**Methods:**

The prospective study conducted from February, 2019 to June, 2020 recruited 312 primigravid pregnant women who reported for antenatal care in three tertiary referral hospitals in northern Ghana. Their socio-demographic, obstetric and adverse foeto-maternal outcome information were obtained with a well-structured questionnaire according to the World Health Organisation (WHO) guidelines. Participants’ blood samples were collected for haematological tests. Odds ratio [OR, 95% confidence interval (CI)] for the association between socio-demographic, obstetric characteristics, foeto-maternal complications and haematological tests in relation to LBW were assessed using logistic regression model.

**Results:**

This study reported a LBW prevalence of 13.5%. Increasing maternal systolic blood pressure (SBP) and diastolic blood pressure (DBP) at 1^st^ visit, before and after delivery significantly increased the odds of LBW. Preterm delivery (PTD<37 weeks) (COR = 9.92, 95% CI (4.87–2020), p<0.001), preeclampsia (PE) (COR = 5.94, 95% CI (2.96–11.94), p<0.001), blood transfusion (COR = 14.11, 95% CI (2.50–79.65), p = 0.003), caesarian delivery (COR = 3.86, 95% CI (1.96–7.58), p<0.001) and male sex neonates (COR = 2.25, 95%CI (1.14–4.47), P = 0.020) presented with increased odds of LBW. Increasing gestational age at delivery presented with 28% reduced odds of LBW (COR = 0.72, 95% CI (1.12–4.40), P = 0.023). Upon controlling for potential confounders in multivariate logistic regression, only gestational age at delivery (AOR = 0.67, 95% CI (0.47–0.96), P = 0.030) remained significantly associated with reduced odds of LBW.

**Conclusion:**

This study found that high blood pressure at 1st visit, before and after delivery results in increased chances of delivering a baby with LBW. Furthermore, PTD<37 weeks, having PE in current pregnancy, and male sex potentiate the risk of LBW. On the other hand, increasing gestational age reduces the risk of LBW. Thus, we recommend that midwives should intensify education to pregnant women on the benefits of regular ANC visits to aid in the early detection of adverse foeto-maternal complications. We also recommend proper clinical management of pregnancies associated with an elevated blood pressure at registration. Also, maternal intrapartum blood pressure measurement could be used to predict LBW in low resourced settings.

## Introduction

Low birth weight (LBW) is defined by the World Health Organization (WHO) as weight at birth less than 2500 g (5.5 lb). LBW is a significant public health problem globally and it is associated with a range of short and long-term consequences. Overall, it is estimated that 15% to 20% of all births worldwide are LBW, representing more than 20 million births per year [1]. LBW is a complex foetal complication which occurs as a result of preterm delivery (PTD<37 weeks), microsomia at term and the overlap between these two complications [2].

In sub-Saharan Africa, risk factors such as high risk pregnancies, younger and advanced maternal age, anaemia during pregnancy as well as hypertensive disorders of pregnancy including gestational hypertension, preeclampsia (PE), eclampsia and poor nutritional status of mother are widely reported to be associated with LBW [3, 4]. Progress made in reducing neonatal mortality has been slower in sub-Saharan Africa compared to any other regions in the world [5].

In 2013, 16.5% of the 11,647 neonates born in the major tertiary referral hospital in Ghana (Korle-Bu Teaching Hospital) had LBW [6]. In 2016, the prevalence of LBW neonates in Ghana was 9.69% [7]. Since then, Ghana has not recorded any significant reduction in LBW [8]. Additionally, about 46% of all LBW babies in Ghana die in the first 28 days of life [9].

Several studies evaluating factors associated with LBW have been conducted in some African countries [10–15]. However, there is paucity of current data in Ghana, particularly in the northern areas of the country where adequate health care is wanting. It is against this background that we prospectively studied the factors associated with LBW in northern Ghana. Findings from the study will provide data which will facilitate the development of policies regarding maternal and foetal health.

## Methods

### Study area

The study was conducted in three major referral (tertiary) hospitals in northern Ghana. The hospitals were the Tamale Teaching Hospital (TTH) in Tamale (Northern Region), the Bolgatanga Regional Hospital (RHB) in Bolgatanga (Upper East Region) and the Bawku Presbyterian Hospital (BPH) in Bawku (Upper East Region). **Table 1** shows the antenatal and delivery information for the three study sites for the year 2019.

**Table 1:** Antenatal and delivery information for the three study sites for the year 2019.

Hospital	Total Deliveries	LBW < 2.5 kg	NBW ≥ 2.5 kg	Vaginal deliveries	CS deliveries
RHB	3245	529	2872	2199	1046
BPH	3251	522	3006	2558	693
TTH	8863	1480	7796	6201	2662

RHB: Bolgatanga Regional Hospital, BPH: Bawku Presby Hospital, TTH: Tamale Teaching Hospital.

### Study design and population

**Fig 1:**
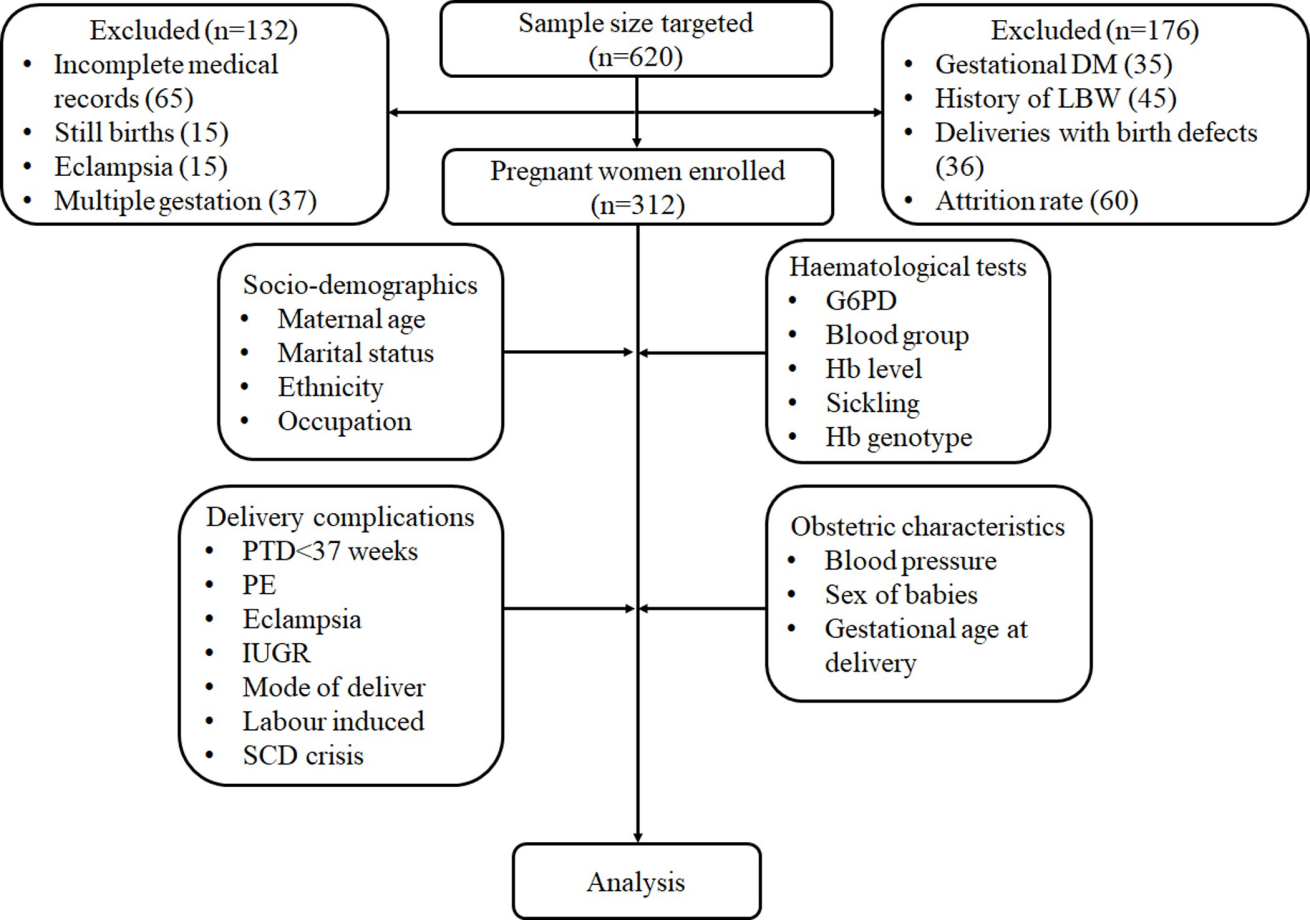
Flowchart of participant selection and analysis.

The study was a prospective multicenter study conducted following the STROBE checklist [16] from February, 2019 to June, 2020. The participants were primigravid pregnant women aged between 15–43 years who reported for antenatal care at the three selected hospitals. The expected sample size was based on the estimated prevalence of LBW (9.69%) [7]. Based on the formula: **n = z**^**2**^**pq/d**^**2**^; where n = minimum sample size, z = confidence limit at 95% = 1.96, p = prevalence of LBW = 0.0969, q = complimentary probability = 1-p, d = precision = 0.05, the minimum sample size required for the study was 134 participants. In this study, 312 pregnant women were recruited. The women included did not have deliveries with birth defect, gestational diabetes, still births, had singleton gestations and did not have a history of LBW (**Fig 1**).

### Obstetric characteristics and perinatal outcomes

The obstetric characteristics evaluated were maternal age, gestational age at delivery and parity. Delivery characteristics such as mode of delivery, maternal blood pressure at booking, before delivery and after delivery were recorded. Adverse maternal outcomes were blood transfusion, labour induction, sickle cell disease (SCD) crisis, preterm delivery (PTD) <37 weeks, PE and eclampsia. Perinatal outcome indicators associated with LBW were sex of new born, birth weight and intrauterine growth retardation (IUGR) (**Fig 1**).

### Blood pressure measurement

Using a mercury sphygmomanometer and a stethoscope, trained personnel measured the blood pressure of participants. Measurements were taken from the left upper arm after the subjects had been sitting for over 5 minutes. Two readings were documented at 15 minutes interval and the average was considered the participant’s blood pressure.

### Anthropometric characteristics

Adhering to WHO standard procedure, a hospital grade electronic weighing scale (China) of capacity 150kg weight and 70~190cm height was used to measure body weight and height respectively, as previously described [17]. Body mass index (BMI) was calculated by dividing weight (kg) by height squared (m^2^).

### Haematological tests

Haematological tests were performed by drawing 5ml of blood from veins located within the antecubital fossa. Assays conducted include ABO/Rh blood grouping, sickling test, haemoglobin electrophoresis, haemoglobin concentration at 1^st^ visit and glucose-6-phosphate dehydrogenase (G6PD) quantitative test. ABO and Rh blood grouping was conducted by utilizing commercially prepared monoclonal anti-A, anti-B, and anti-D antisera (Agappe Diagnostics Ltd., India) according to Cheesbrough protocol [18]. Haemoglobin levels were determined using the Sysmex KX-21 N Automated Hematology Analyzer (Sysmex Corporation Kobe, Japan). Haemoglobin variants determined were AA, AS, SS & SC genotypes. This was done at an alkaline pH of 8.5 (Beijing Liuyi Instrument Factory, China).

G6PD status was determined quantitatively (RANDOX, UK). Study participants were classified as: G6PD Normal if G6PD activity was ≥ 4000 mU/gHb (≥4.00 U/gHb), G6PD Partial Defect if G6PD activity was 1000 to 3999 mU/gHb (1.00–3.99 U/gHb) and G6PD full defect if G6PD activity was <1000 mU/gHb (<1.00 U/gHb) [19].

### Sources of data

Participant antenatal and delivery information outlining socio-demographic, obstetric characteristics and perinatal outcomes were obtained by trained interviewers (midwives) through structured questionnaires at time of study inclusion, at delivery and 48 hours postpartum.

### Outcome

Birth weight in grams (g) was measured within 24 hours after birth and LBW was diagnosed if a neonate weighed less than 2500g [20].

### Statistical analysis

Categorical variables were presented as frequencies and percentages. Continuous data were presented as mean ± SD. Logistic regression analysis was used to evaluate the risk factors associated with LBW. To determine potential factors associated with birth weight, we first performed univariate logistic regression analysis. This was followed by multivariate logistic regression analysis using the enter method for variables with p-values <0.05 after univariate analysis to identify independent risk factors. Statistical analysis was performed using Stata version 14.2, GraphPad Prism 8 and MedCalc statistical software version 19.8. The receiver operating characteristics (ROC) curve was computed at an estimated prevalence of LBW, 9.69% and confidence interval (CI) of 95%. All tests were two-sided and p-value < 0.05 was considered statistically significant.

### Ethics approval and consent to participate

Ethical approval for this study was obtained from the institutional review board of the Navrongo Health Research Centre of the Ghana Health Service with approval number NHRCIRB326. Written informed consent was obtained from all participants who opted to participate after the aims and objectives of the study had been explained to them. Consent for minors were obtained from the parents/guardians. Participation was voluntary, and respondents were assured that the information obtained was strictly for research and academic purposes only and were guaranteed the liberty to opt out from the study at their own convenience.

## Results

Antenatal and delivery information for the three sites for the year 2019 is represented in **Table 1**.

### General characteristics of participants

A total of 312 pregnant women with average age and BMI of 27.22 ± 6.08 years old and 25.44 ± 6.79 kg/m^2^ respectively, were enrolled in the study. A higher proportion of the women enrolled in the study were of Northern ethnicity (278/312 vs. 34/312), were married (91.67%) and nulliparous (38.46%), had O positive blood group (33.94%), were of haemoglobin genotype AA (82.69%) and anaemic defined by haemoglobin level <11 g/dl at 1^st^ ANC visit (53.85%). Majority of the participants did not deliver prematurely (96.15%), did not have PE (92.31%) and eclampsia (96.89%) in the current pregnancy. Majority of neonates had an appearance, grimace, activity and respiration (APGAR) score >7 at 1 minute (87.82%) and 5 minutes (94.23%), respectively (**Table 2**).

**Table 2:** Sociodemographic and clinical characteristics of participants.

Variable	n (%)
**Maternal age**	
(mean ± SD)	27.22 ± 6.08
15–19	28(8.97)
20–29	176(56.41)
30–34	60(19.23)
35–45	48(15.38)
**Ethnicity**	
Northerner	278(89.10)
Southerner	34(10.90)
**Marital status**	
Married	286(91.67)
Single	20(6.41)
Divorced	6(1.92)
**Occupation**	
Civil servant	116(37.18)
Self-employed	114(36.54)
Unemployed	82(26.28)
**Blood pressure measurements** **(mmHg)**	
SBP 1^st^ visit	110.2 ± 19.76
DBP 1^st^ visit	68.88 ± 12.53
SBP at delivery	126.4 ± 19.57
DBP at delivery	80.03 ± 14.99
SBP 48 hours postpartum	119.7 ± 16.17
DBP 48 hours postpartum	74.28 ± 14.48
**Baby sex**	
Female	156(50.0)
Male	156(50.0)
**Parity**	
Nulliparity	120(38.46)
Uniparity	74(23.72)
Multiparity	118(37.82)
**Anthropometry**	
BMI (mean ± SD)	25.44 ± 6.79
**APGAR at 1 minute**	
<7	38(12.18)
>7	274(87.82)
**APGAR at 5 minutes**	
<7	18(5.77)
>7	294(94.23)
**Hb genotype**	
AA	258(82.69)
AS	40(12.82)
SC	12(3.85)
SS	2(0.64)
**Blood group**	
A negative	12(3.85)
A positive	62(19.87)
AB positive	24(7.69)
B positive	80(25.64)
O negative	10(3.21)
O positive	124(33.97)
**Sickling**	
Negative	258(82.69)
Positive	54(17.31)
**1st Trimester Hb**	
<11g/dl	168(53.85)
>11g/dl	144(46.15)
**G6PD status**	
no defect	274(87.82)
full defect	26(8.33)
partial defect	12(3.85)
**Respiratory distress**	
No	292(93.59)
Yes	20(6.41)
**PTD <37 weeks**	
No	248(79.49)
Yes	64(20.51)
**PE in current pregnancy**	
No	216(69.23)
Yes	96(30.77)
**Eclampsia in current pregnancy**	
No	302(96.79)
Yes	10(3.21)
**Labour induced**	
No	300(96.15)
Yes	12(3.85)
**IUGR**	
No	310(99.36)
Yes	2(0.64)
**Blood transfused**	
No	306(98.08)
Yes	6(1.92)
**SCD crisis**	
No	306(98.08)
Yes	6(1.92)
**Mode of delivery**	
Caesarian section	106(33.97)
Vaginal delivery	206(66.03)

Categorical variables are presented as number with percentage in parenthesis. SCD; sickle cell disease, PE; preeclampsia, IUGR; intrauterine growth restriction, G6PD; glucose-6-phosphate dehydrogenase, PTD; preterm delivery.

**Fig 2:**
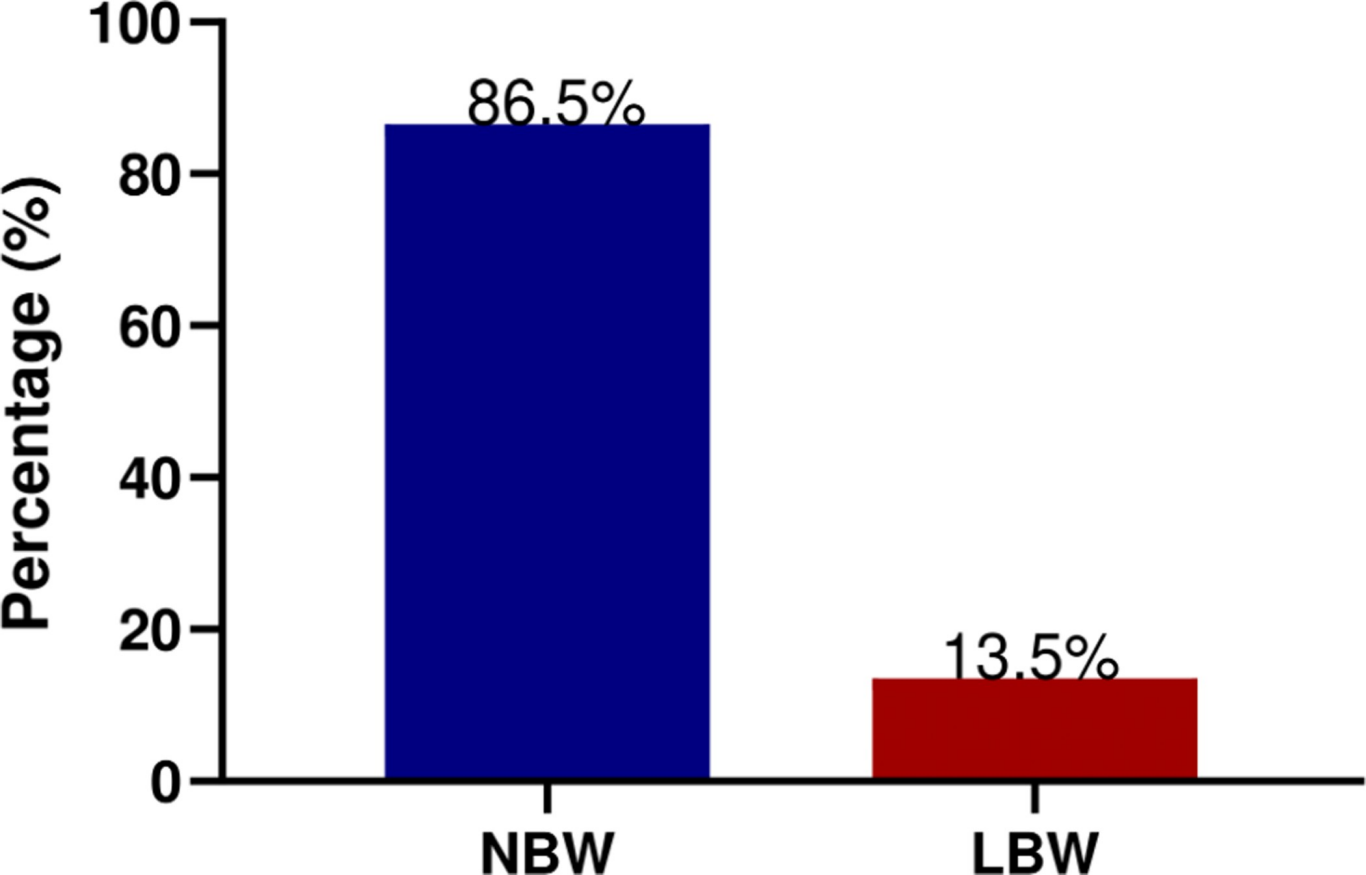
Prevalence of low birth weight.

Among the 312 participants, the prevalence of Normal birth weight (NBW) and Low birth weight (LBW) were 86.5% (270/312) and 13.5% (42/312), respectively (**Fig 2**).

### Crude (COR) and adjusted odds ratio (AOR) for factors potentially associated with LBW

Pregnant women with PTD <37 weeks (COR = 9.92, 95% CI (4.87–20.20), p<0.001), PE in current pregnancy (COR = 5.94, 95% CI (2.96–11.94), p<0.001), participants who had blood transfusion (COR = 14.11, 95% CI (2.5–79.65), p = 0.003), male sex neonates (COR = 2.25, 95% CI (1.14–4.47), p = 0.020) and caesarian section (COR = 3.86, 95% CI (1.96–7.58), p<0.001) presented with significantly higher odds of LBW. Increasing gestational age at delivery presented with 28% reduced odds of LBW (COR = 0.72, 95% CI (0.64–0.80), p<0.001). Additionally, a unit increase in systolic blood pressure (SBP) and diastolic blood pressure (DBP) at 1^st^ visit, before delivery and post-delivery presented with significantly higher odds of LBW. Upon controlling for potential confounders in multivariate logistic regression, only gestational age at delivery (AOR = 0.67, 95% CI (0.47–0.96), p = 0.030) remained significantly associated with reduced odds of LBW (**Table 3**).

**Table 3:** Crude and multivariate logistic regression of factors associated with LBW.

Variable	COR(95%CI)	p-value	AOR(95%CI)	p-value
**Maternal age (years)**				
15–19	1			
20–29	2.05(0.46–9.21)	0.348		
30–34	3.25(0.68–15.64)	0.141		
35–45	1.18(0.20–6.91)	0.853		
**Ethnicity**				
Northerner	1			
Southerner	1.44(0.56–3.72)	0.451		
**Marital status**				
Married	1			
Single	1.63(0.52–5.14)	0.403		
Divorced	-	-		
**Occupation**				
Civil servant	1			
Self-employed	1.02(0.46–2.25)	0.961		
Unemployed	1.50(0.67–3.34)	0.322		
**Blood pressure measurements** **(mmHg)**				
SBP 1^st^ visit	1.03(1.02–1.05)	**<0.001**	1.00(0.93–1.07)	0.997
DBP 1^st^ visit	1.06(1.03–1.08)	**<0.001**	0.98(0.88–1.10)	0.753
SBP at delivery	1.06(1.04–1.08)	**<0.001**	1.03(0.97–1.10)	0.343
DBP at delivery	1.07(1.04–1.09)	**<0.001**	1.04(0.96–1.13)	0.308
SBP 48 hours postpartum	1.04(1.01–1.06)	**0.001**	1.02(0.96–1.08)	0.597
DBP 48 hours postpartum	1.06(1.03–1.10)	**<0.001**	0.98(0.92–1.05)	0.642
**Gestational age at delivery**				
Gestational age at delivery	0.72(0.64–0.80)	**<0.001**	0.67(0.47–0.96)	**0.030**
**Baby sex**				
Female	1			
Male	2.25(1.14–4.47)	**0.020**	3.81(0.93–15.63)	**0.063**
**Parity**				
Nulliparity	1			
Uniparity	1.26(0.56–2.83)	0.579		
Multiparity	0.88(0.41–1.88)	0.733		
**Hb-genotype**				
AA	1			
AS	0.32(0.08–1.40)	0.132		
SC	3.08(0.88–10.77)	0.078		
SS	1	-		
**Blood group**				
A negative	1			
A positive	0.48(0.18–1.26)	0.974		
AB positive	1.13(0.49–2.63)	0.772		
B positive	1.18(0.36–3.85)	0.787		
O negative	1.47(0.29–7.5)	0.641		
O positive	1.18(0.24–5.82)	0.841		
**Sickling status**				
Negative	1			
Positive	0.77(0.31–1.93)	0.579		
**1st Trimester Hb**				
<11g/dl	1			
≥11g/dl	1.07(0.56–2.05)	0.838		
**G6PD status**				
no defect	1			
full defect	-	-		
partial defect	-	-		
**PTD<37 weeks**				
No	1		1	
Yes	9.92(4.87–20.20)	**<0.001**	2.56(0.24–27.67)	0.440
**PE in current pregnancy**				
No	1		1	
Yes	5.94(2.96–11.94)	**<0.001**	0.13(0.01–1.90)	0.137
**Eclampsia in current pregnancy**				
No	1			
Yes	1.64(0.34–7.99)	0.542		
**Labour induced**				
No	1			
Yes	1.3(0.27–6.15)	0.741		
**IUGR**				
No	1			
Yes	-			
**Blood transfused**				
No	1		1	
Yes	14.11(2.5–79.65)	**0.003**	2.77(0.1–80.51)	0.553
**SCD crisis**				
No	1			
Yes	3.33(0.59–18.75)	0.173		
**Mode of delivery**				
Vaginal delivery	1		1	
Caesarian section	3.86(1.96–7.58)	**<0.001**	4.13(0.99–17.24)	**0.051**
**Anthropometry**				
BMI	1.02(0.97–1.07)	0.405		

SCD; sickle cell disease, PE; preeclampsia, IUGR; intrauterine growth restriction, G6PD; glucose-6-phosphate dehydrogenase, BMI; body mass index, PTD; preterm delivery, Hb; haemoglobin, SBP; systolic blood pressure, DBP; diastolic blood pressure, COR; crude odds ratio, AOR; adjusted odds ratio.

### Diagnostic performance of systolic and diastolic blood pressure measurements at study inclusion, at delivery and 48 hours post-delivery in predicting LBW

**Fig 3:**
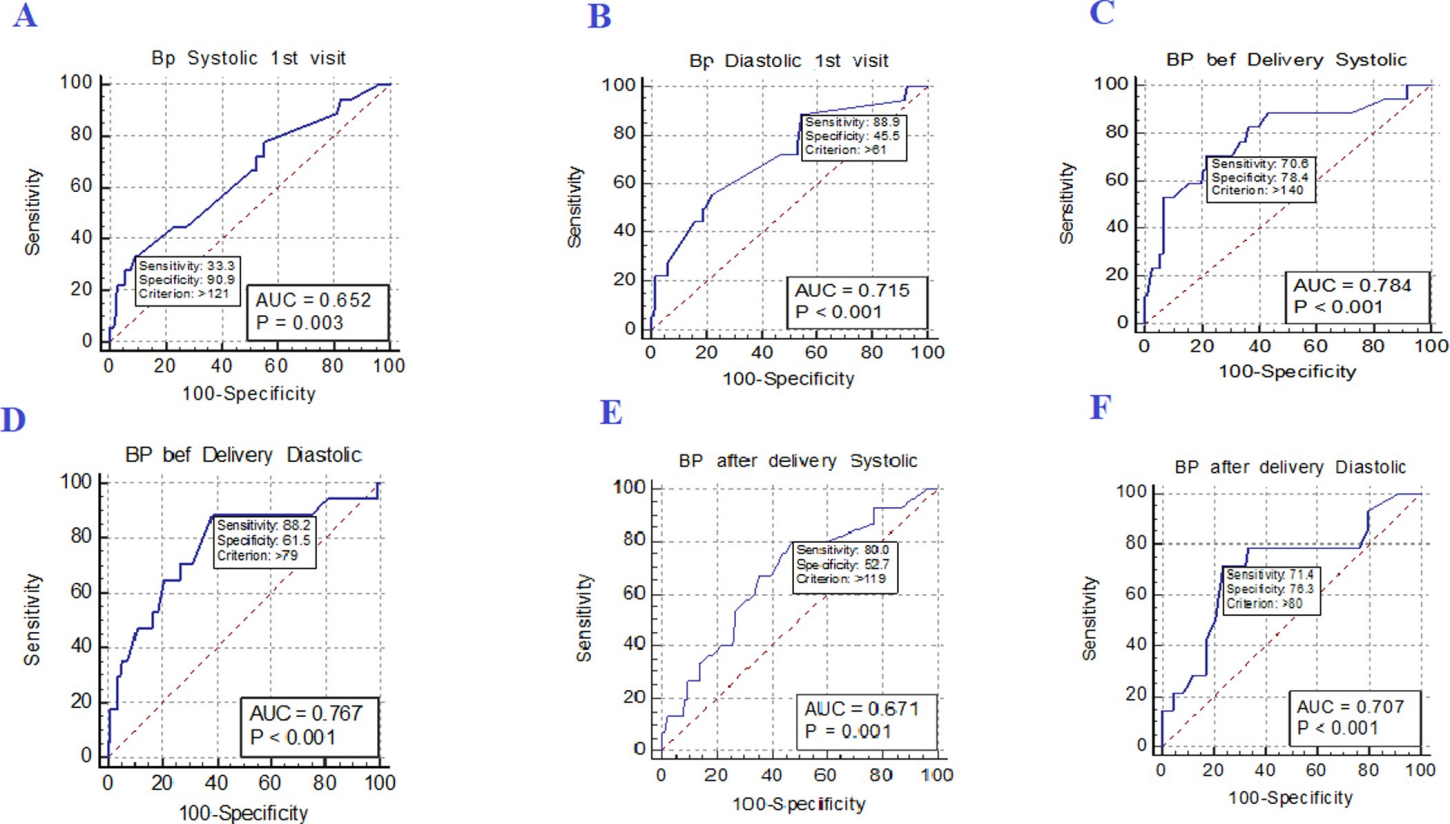
Diagnostic performance of blood pressure measurements in predicting LBW at study inclusion, at delivery and 48 hours post-delivery. (A) SBP at 1^st^ visit, (B) DBP at 1^st^ visit, (C) SBP before delivery, (D) DBP before delivery, (E) SBP after delivery, (F) DBP after delivery.

DBP at 1^st^ ANC visit, and at delivery and SBP at 48 hours post-delivery presented with excellent sensitivities (88.90%, 88.24% and 80.00%, respectively) and a good to moderate Area under the curve (AUC) (0.715(0.660–0.765), 0.767(0.709–0.817) and 0.671 (0.604–0.734), respectively), in predicting LBW. SBP at 1^st^ visit had the lowest sensitivity (33.33%) but the highest specificity (90.91%) followed by SBP at delivery (78.38%). DBP at 1^st^ visit was the least specific (45.00%) in predicting LBW (**Fig 3** and **Table 4**).

**Table 4:** Diagnostic performance of systolic and diastolic blood pressure measurements at study inclusion, at delivery and 48 hours post-delivery in predicting LBW.

Blood pressure measurements	Cut-off (mmHg)	Sensitivity (95% CI)	Specificity (95% CI)	PPV (%)	NPV (%)	AUC(95% CI)
SBP 1^st^ visit	>121	33.33(18.6–51.0)	90.91(86.8–94.1)	28.2	92.7	0.652(0.595–0.706)
DBP 1^st^ visit	>61	88.90(73.9–96.9)	45.50(39.3–51.7)	14.9	97.4	0.715(0.660–0.765)
SBP at delivery	>140	70.59(52.5–84.9)	78.38(72.4–83.6)	25.9	96.1	0.784(0.728–0.833)
DBP at delivery	>79	88.24(72.5–96.7)	61.50(54.7–68.0)	91.7	98.0	0.767(0.709–0.817)
SBP 48 hours postpartum	>119	80.00(61.4–92.3)	52.69(45.3–60.0)	15.4	96.1	0.671 (0.604–0.734)
DBP 48 hours postpartum	>80	71.43(51.3–86.8)	76.34(69.6–82.3)	24.5	96.1	0.707(0.641–0.767)

The receiver operating characteristics (ROC) curve was used to evaluate the diagnostic performance of systolic and diastolic blood pressure measurements at study inclusion, delivery and 48 hours post-delivery in relation to LBW. SBP: systolic blood pressure, DBP: diastolic blood pressure, AUC: area under the curve, NPV: negative predictive value, PPV: positive predictive value, mmHg: millimetres mercury and CI: confidence interval.

## Discussion

The prevalence of LBW in this study was 13.5%. This prevalence is not very different from previously reported prevalence from similar studies conducted in southern part of Ghana by the United Nations Children’s Fund (UNICEF) and in northern Ghana by Agoringya et al. who correspondingly reported 13.0% and 13.8% prevalence, respectively [6, 21]. Contrarily, in the Brong Ahafo region, another region in the south of Ghana, Zackariah et al. reported a LBW prevalence of 11% which is lower than the prevalence reported in our study [8]. Furthermore, varying prevalence rates have been reported in other African countries. In Zimbabwe, Feresu et al. found a LBW prevalence of 16.7% [3]. Mengesha et al. reported a prevalence of 10.5% in Ethiopia [13]. In Northern Ethiopia, Gebregzabiherher et al. found a LBW prevalence of 10% [22]. Oladeinde et al. in Nigeria found a LBW prevalence of 6.3% [23]. Whereas, the prevalence of LBW in Togo in 2015 was 16.1% [24]. In 2018, a study by Zhifei et al. found LBW prevalence of 10.2% in Ghana and prevalence of 13.4%, 12.1%, 15.7% and 10% in Burkina Faso, Malawi, Senegal and Uganda, respectively [24].

In Ghana and sub-Saharan Africa, there is paucity of data on the association between birth weight and maternal blood pressure. The blood pressure measurements presented with a good predictive power for LBW. Our finding suggests that in low resourced settings, elevation in maternal blood pressure measurements at 1^st^ visit and delivery could serve as an inexpensive predictor of LBW. In contrast, Hovi et al. in Finland and Taylor et al. in Great Britain found no significant association between maternal high blood pressure and LBW [25, 26]. These findings show that additional studies on the potential association between LBW and maternal blood pressure is required.

In this study, PE was associated with increased odds of LBW. This finding could be due to the mechanistic relationship PE has with birth weight. An abnormally implanted placenta which predisposes a woman to PE is also implicated in hypo-perfusion culminating in hypoxia which restricts fetal growth leading to low birth weight [27, 28]. The findings concurs with the findings of Bonsaffoh et al. in Ghana who showed a strong association between PE and adverse perinatal outcomes such as LBW, respiratory distress, intensive care unit admission and intrauterine growth restriction [29]. In Tanzania, Mitao and colleagues also reported a relationship between LBW and several risk factors such as PE, eclampsia, chronic hypertension, caesarian section and low maternal BMI [14].

First trimester haemoglobin concentration of pregnant women who delivered LBW babies were not significantly different from those who delivered NBW babies in this study. This is because the anaemic condition at this stage of pregnancy is often resolved with the administration of haematinics or by dietary modifications. This finding is in variance with the findings of Vanek and his colleagues in Israel who outlined an association between hypertensive disorders of pregnancy and several risk factors such as blood transfusions, labour induction, hydramnios and gestational diabetes resulting in low birth weight [30].

We also found caesarian delivery and PTD<37 as risk factors for LBW. Our study agrees with Chen et al., who found LBW to be associated with maternal and fetal complications necessitating caesarian delivery in avoidance of intrauterine foetal demise, still birth and neonatal death at term [31]. Chronic intrauterine hypoxia and growth restriction characterizes LBW as a result of an impaired oxygen and nutrient transfer from maternal compartment to foetus. Furthermore, increasing gestational age at delivery (in weeks) consistently reduced the odds of LBW even after multivariate adjustment. The mechanism underlying the birth of premature neonates still needs further scientific investigations. We hypothesise that preterm infants are mostly underweight since they do not have adequate time for optimum intrauterine fetal growth. As a result, term delivery results in normal birth weight. Consistent with our study findings is a WHO multi-country survey on maternal and new born health by Ota et al, who related preterm deliveries (early deliveries) to gestational hypertensions, PE and eclampsia [32]. This is because complications associated with hypertensive disorders of pregnancies resolve only after delivery of the placenta hence caesarean delivery before term is the best option resulting in neonates with LBW. Furthermore, similar studies have related preterm delivery to LBW due to subclinical infections culminating in membrane rupture, to micronutrient deficiencies and placental abnormalities as a result of increased serum alpha-fetoprotein levels [33–35].

Our study found the male sex to be associated with higher odds of LBW. On the contrary, Tshotetsi et al., in South Africa and Ayuurebobi et al., in Ghana could not find an association between male sex and LBW [36, 37]. Similar studies found the male sex to be associated with higher risk for LBW [38, 39]. Roy et al. in India found the male sex to be disadvantaged due to its poor perinatal outcomes and higher risk of LBW leading to septicaemia and neonatal demise [38]. Stevenson et al., in the United States also found a higher mortality and postnatal complications in very LBW males than in females [39]. Higher proportions of these male neonates in their cohort had lower Apgar scores, respiratory distress syndrome or lung related injuries and disabilities, and were generally less stable than female neonates.

### Strength and limitation of the study

The study included several risk factors for LBW; socio-demographic, obstetric characteristics, haematological tests and foeto-maternal complications of pregnancy in one study. Additionally, the relationship between foeto-maternal complications of pregnancies such as PE, PTD<37 weeks, blood transfusion, caesarian deliveries and LBW were evaluated. Our study is limited due to the small sample size. Additionally, a substantial number of pregnant women who deliver in the 3 tertiary hospitals used for the study typically register for antenatal care elsewhere and are referred to tertiary hospitals to deliver when there is any suspicion of possible complications. Hence, care should be taken when interpreting the data since the results may not be generalizable to the entire population

## Conclusion

This study found that high blood pressure at 1^st^ visit, before and after delivery results in increased chances of delivering a baby with LBW. Furthermore, PTD<37 weeks, having PE in current pregnancy, male sex and blood transfusion potentiate the risk of LBW. On the other hand, increasing gestational age reduces the risk of LBW. Thus, we recommend that midwives should intensify education to pregnant women on the benefits of regular ANC visits to aid in the early detection of adverse foeto-maternal complications. We further recommend proper clinical management of pregnancies associated with an elevated blood pressure at registration. Also, maternal intrapartum blood pressure measurement could be used to predict LBW in low resourced settings.
